# Severe Electrolyte Disturbances Complicated by Seizures and Acute Kidney Injury Within 10 Days of Starting Indapamide

**DOI:** 10.7759/cureus.11303

**Published:** 2020-11-02

**Authors:** Mohammed A Alamin, Ashraf Ahmed, Aasir M Suliman

**Affiliations:** 1 Internal Medicine Department, Hamad General Hospital, Hamad Medical Corporation, Doha, QAT

**Keywords:** indapamide, thiazides, electrolyte disturbances, hyponatremia, hypokalemia, acute kidney injury, rhabdomyolysis, seizures

## Abstract

Indapamide is one of the most effective and well-known anti-hypertensive medications. Electrolyte disturbances have been classically recognized as a typical side effect profile of indapamide. The most common electrolyte imbalance described with indapamide was hypokalemia; however, hyponatremia is being increasingly reported. In this case, we report a unique form of severe electrolytes derangement (hyponatremia, hypokalemia, hypophosphatemia, and hypocalcemia), which was complicated by seizures, rhabdomyolysis, and acute kidney injury that occurred within only 10 days of indapamide initiation. The patient was admitted to the medical intensive care unit for prompt electrolyte replacement and close monitoring. With the discontinuation of indapamide and the prompt replacement of the deficient electrolytes, the patient’s condition has improved dramatically, and he was discharged in a good state of health. Electrolyte disturbances are expected to be seen with indapamide usage, and it might be associated with severe consequences like arrhythmias and seizures. This case report would raise awareness and add to the importance of closely following patients after prescribing indapamide.

## Introduction

Hyponatremia (serum sodium < 136 mEq/L) is the most common electrolyte imbalance observed in clinical practice. Among the various causes of drug-induced hyponatremia, thiazide-induced hyponatremia comprises a leading cause, while loop diuretics only occasionally induce hyponatremia [1]. Indapamide is a thiazide-like diuretic that has an identical physiological activity to thiazide diuretics. It exerts its action mainly on the distal convoluted tubule (DCT) by enhancing the execration of sodium, chloride, and water. Indapamide is known to cause electrolyte disturbances, mainly hyponatremia and hypokalemia [2-4]. The investigation of possible thiazide-associated hyponatremia includes the exclusion of other causes of decreased sodium levels and the identification of the characteristics of hyponatremia due to thiazides (extracellular volume depletion-related or syndrome of inappropriate antidiuretic hormone secretion (SIADH)-like). In this report, we present a case of indapamide-induced severe electrolyte disturbances along with seizure complicated by rhabdomyolysis and acute kidney injury with a literature review.

## Case presentation

The patient is a 52-year-old Indian gentleman with a past medical history of hypertension for more than 20 years; he was on bisoprolol and amlodipine, compliant to his medications. Upon review at a private clinic, his blood pressure (BP) was found to be uncontrolled, for which he has prescribed indapamide 2.5 mg 10 days prior to presentation. The patient was brought by ambulance to the emergency department (ED) because of a decreased level of consciousness. At the scene, the emergency medical services (EMS) team found the patient confused and disoriented, with a Glasgow Coma Scale (GCS) of 13/15. His co-workers did not witness any abnormal movements.

In the ED, the patient was found to be confused with GCS 14/15; tongue bite mark and urine in his clothes were noted. His temperature was 36.9 °C, heart rate (HR) 69 beat/minute, BP 156/88 mmHg, respiratory rate (RR) 19 breath/minute, oxygen saturation 96% on room air. Physical examination, including cardiac and respiratory systems, was unremarkable. While in ED, the patient developed an episode of generalized tonic-clonic seizures, with tongue bite and urinary incontinence, which was alleviated by 2mg intravenous (IV) lorazepam, after which his GCS dropped to 10 (Eye 4. Motor 4. Verbal 2). 

ECG was done and showed U wave in the anterior chest leads, QTc 480 milliseconds (ms) (average < 500 ms), QRS was 130 ms (average 80-100 ms). Lab investigations were done (Table 1); he was found to have severe hyponatremia, hypokalemia, hypochloremia, hypophosphatemia, acute kidney injury (AKI), elevated myoglobin and creatine kinase (CK). Plain computed tomography (CT) of the head was done and found to be unremarkable. Chest x-ray was normal. The patient was admitted to the intensive care unit (ICU) for which a central line was inserted for intravenous hypertonic saline and potassium replacement. His condition improved clinically and biochemically, and he was stepped down to the medical ward after two days. The patient observed over another couple of days and then discharged in a good clinical condition, he was asymptomatic; his sodium level was 128 mmol/L, potassium 3.7 mmol/L, creatinine was 108 umol/L. The patient was reviewed within one week after discharge at the outpatient clinic; his sodium level was 132 mmol/L, potassium 3.7 mmol/L, and creatinine was 111 umol/L.

**Table 1:** Lab Investigations on Admission: WBC: white blood cells, Hb: hemoglobin, TSH: thyroid stimulating hormone, T4: thyroxine

Variable	Value	Reference Range
WBC	11.4 x10^3/uL	4-10 x10^3/uL
Hb	16.9 gm/dl	13-17 gm/dl
Platelets	165 x10^3/uL	150 – 400 x10^3/uL
Urea	9.3 mmol/L	2.76 – 8.07 mmol/L
Creatinine	187 umol/L	62 – 106 umol/L
Sodium	100 mmol/L	136 – 145 mmol/L
Potassium	1.8 mmol/L	3.5 – 5.1 mmol/L
Chloride	<60 mmol/L	98-107 mmol/L
Bicarbonate	28 mmol/L	22 - 29 mmol/L
Corrected Calcium	2.17 mmol/L	2.15 – 2.50 mmol/L
Phosphorus	0.44 mmol/L	0.81 – 1.45 mmol/L
Magnesium	0.74 mmol/L	0.66 – 1.07 mmol/L
Creatine Kinase	>22000 U/L	39 - 308 U/L
Myoglobin	6,821 ng/mL	28 - 72 ng/mL
Serum Osmolality	221 mmol/kg	275 - 295 mmol/kg
Urine Osmolality	288 mmol/kg	150 – 1,150 mmol/kg
Spot Urine Sodium	67 mmol/L	N/A
Spot Urine potassium	21.1 mmol/L	N/A
Spot Urine Chloride	61 mmol/L	N/A
Ethanol	<2.2 mmol/L	Critical High > 44.1 mmol/L
TSH	0.37 mIU/L	0.30 – 4.20 mIU/L
Free T4	17.2 pmol/L	11.6 – 21.9 pmol/L
Random Cortisol level	440 nmol/L	133 - 537 nmol/L (AM)

## Discussion

Indapamide-induced severe symptomatic hyponatremia and hypokalemia are reported in the literature, and it is presumed to be mainly due to its direct natriuretic effect; however, indapamide-induced syndrome of inappropriate antidiuretic hormone (SIADH) is reported as a mechanism of hyponatremia, mainly in the elderly population [5]. Indapamide-induced hyponatremia seems to be reversible and tends to improve after stopping the drug; however, re-challenging the patients again with indapamide even at lower doses and frequencies is not advised, as it was reported to produce the same notorious side effect again [6]. There are no hints toward which patients are going to develop electrolyte disturbances; however, low body weight, elderly age, and physical immobility are generally known to predispose to diuretic-induced electrolyte derangement [7].

As electrolyte disturbance was mostly reported to be hyponatremia and hypokalemia, the predominance presentation was secondary to the severe hyponatremia rather than hypokalemia in the form of neurological symptoms, mainly delirium, unsteady gaits, recurrent falls, along with nausea and vomiting. Seizures have been reported as well, but they were self-limited and did not require sedation or antiepileptic drugs [8-10]. Mok et al. reported a unique cardiac case of acquired Brugada syndrome and prolonged QT interval that developed polymorphic ventricular arrhythmia and cardiac arrest due to severe hyponatremia and severe hypokalemia secondary to indapamide [11]. In addition to this, indapamide is reported to alter phosphate metabolism and hypophosphatemia, which is presumed to be mainly due to renal phosphorus loss [12]. Also, indapamide is reported to cause a direct acute kidney injury and renal failure; a study in French National Pharmacovigilance Database has reported 11 patients who developed acute kidney injury secondary to indapamide, where two of them required renal replacement therapy [13].

To our knowledge, indapamide-induced severe hyponatremia that provokes generalized tonic-clonic seizures complicated by myonecrosis and acute kidney injury is not reported in the literature. What is more, the other accompanying electrolyte derangements (hypokalemia, hypophosphatemia, and hypocalcemia) were also unique in this patient, and the short duration of developing the electrolyte disturbance (within 10 days) following initiation of indapamide.

## Conclusions

In conclusion, physicians should be alert when prescribing indapamide, and the basal metabolic panel should be strictly followed up in short intervals, as electrolyte disturbances might develop within a few days after initiating the treatment. In case it develops, prompt stopping of the drug is necessary, and it needs to be kept in the patients’ medical records as prescribing the drug again will likely produce the same side-effect. The sequelae of electrolyte disturbances are unpredictable and may range from being asymptomatic, up to developing seizures and arrhythmias, which might cause morbidity and end-organ damage.
